# Supplementary material for: RSV can infect the human nasal epithelium via the basolateral route and shows distinct subgroup infectivity and basal cell tropism
Source: J Virol. 2026 Jun 25;100(7):e00374-26. doi: 10.1128/jvi.00374-26 (PMC13386820; doi:10.1128/jvi.00374-26)
Supplement: Supplemental material — Table S1; Fig. S1 to S8. [file jvi.00374-26-s0001.pdf]

Supplemental Table 1

**INFANT HNO-ALIs**		
**Sample ID**	**Gender**	**Age range**
HNO9002	F	12-24 months
HNO9003	F	<12 months
HNO9004	F	<12 months
HNO9005	M	12-24 months
HNO9006	M	12-24 months
HNO9007	F	<12 months
HNO9008	M	12-24 months
HNO9009	M	12-24 months
**ADULT HNO-ALIs**		
**Sample ID**	**Gender**	**Age range**
HNO204	F	65+ years
HNO02	M	50-64 years
HNO918	F	18-45 years
HNO919	F	18-45 years
HNO923	M	18-45 years
HNO929	M	65+ years
HNO930	M	65+ years
HNO934	F	50-64 years

Supplemental Table 1: Demographics of adult and infant HNO-ALI donors

Supplemental Figure 1

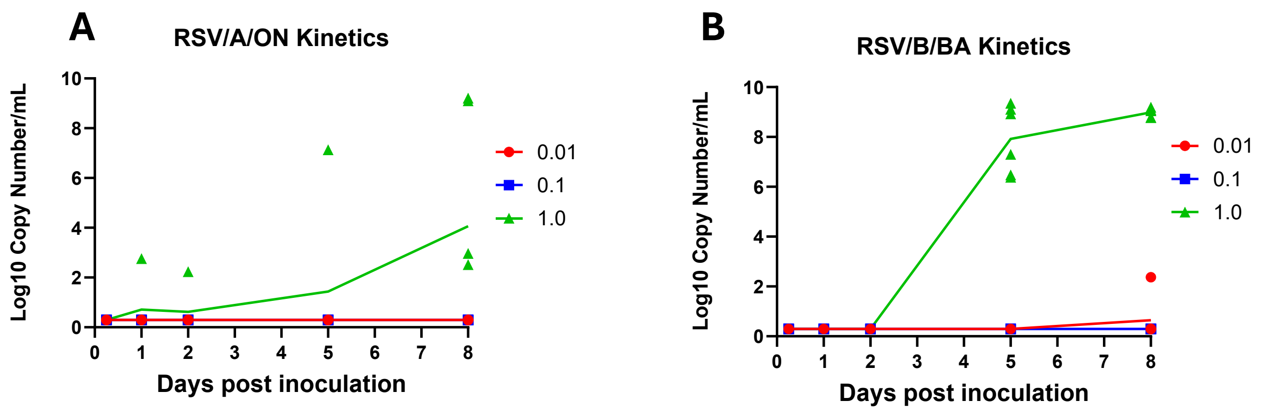

Supplemental Figure 1: Viral RNA detection in apical lumen with increasing multiplicity of infection (MOI). qPCR of apical viral RNA with basolateral (A) RSV/A/ON and (B) RSV/B/BA inoculation at different MOIs. Data is pooled from one adult and one infant line.

Supplemental Figure 2

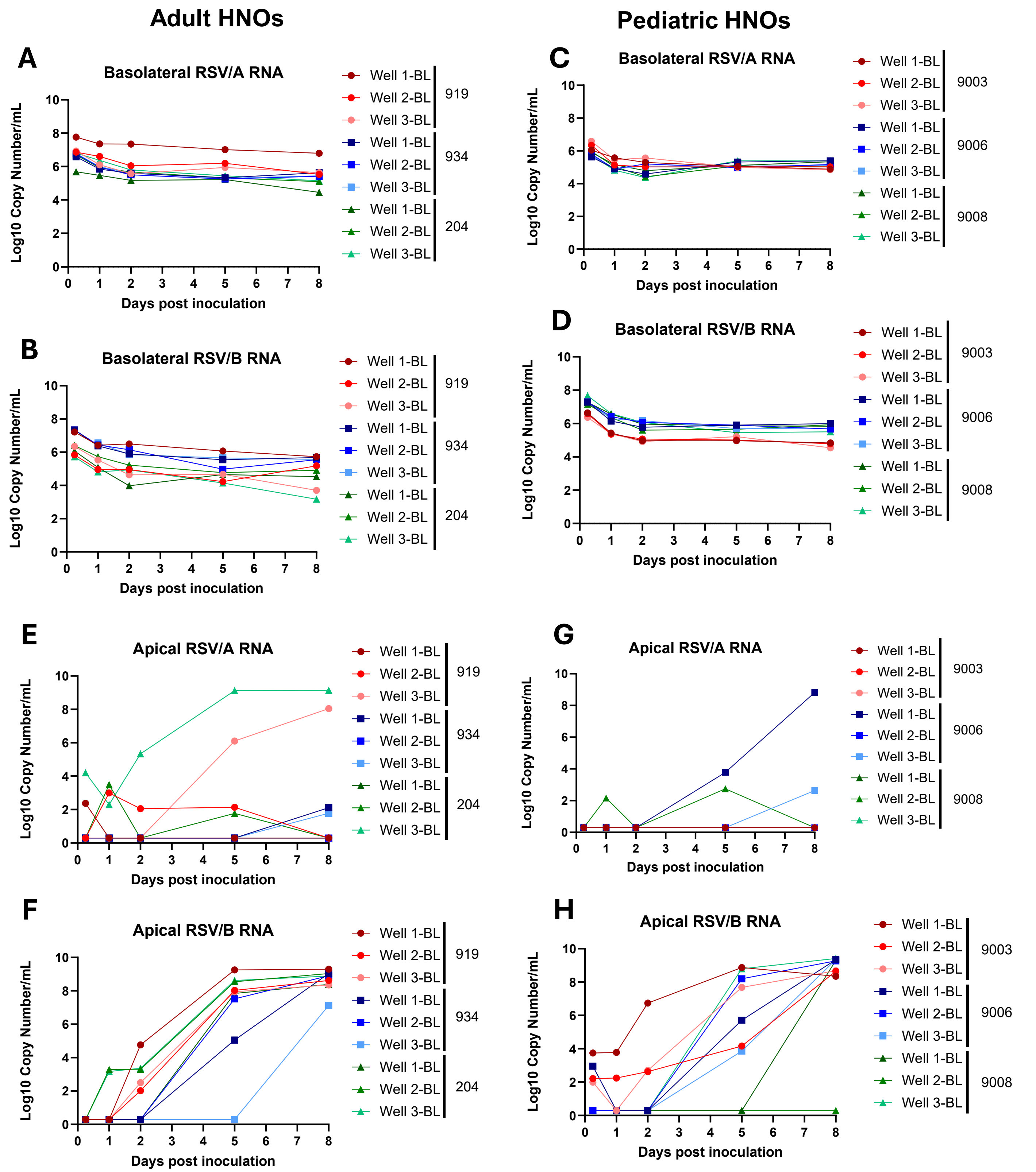

Supplemental Figure 2: Viral RNA detection in basolateral media and apical lumen with basolateral inoculation of RSV/A/ON and RSV/B/BA. qPCR of (A-D) basolateral and (E-H) apical viral RNA with basolateral RSV/A/ON and RSV/B/BA inoculation in three technical replicates from three representative adult (919, 934, 204) lines (left) and three representative infant (right) HNO-ALI cultures (9003, 9006, 9008).

Supplemental Figure 3

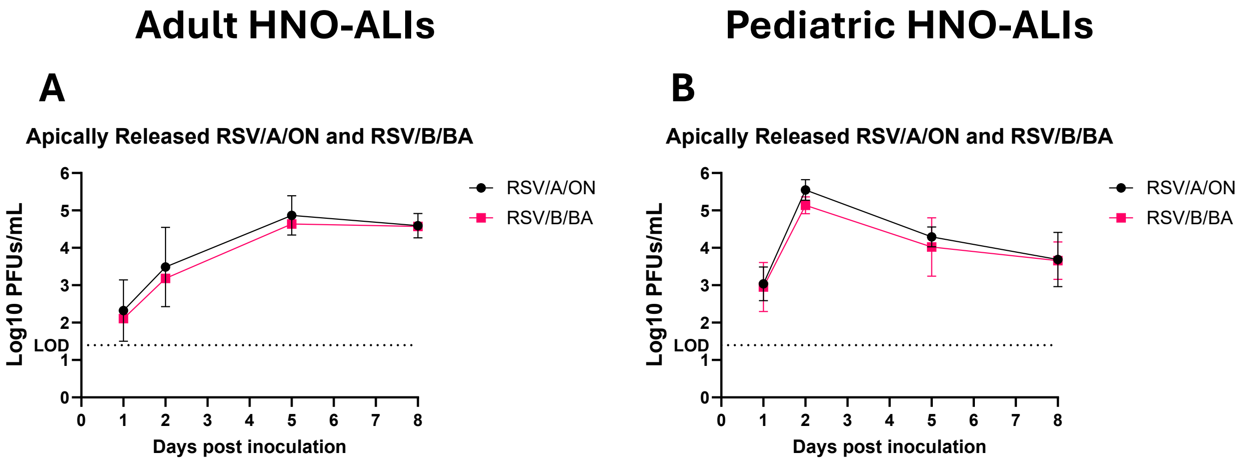

Supplemental Figure 3: Viral kinetics of apical RSV/A/ON and RSV/B/BA infection. Pooled plaque assay data for (A) RSV/A/ON and (B) RSV/B/BA apical infection of adult (n=4 lines) and infant (n=4 lines) HNO-ALI cultures. LOD = limit of detection. Data represent mean ± standard deviation. Adapted from *Aloisio et al* (12).

Supplemental Figure 4

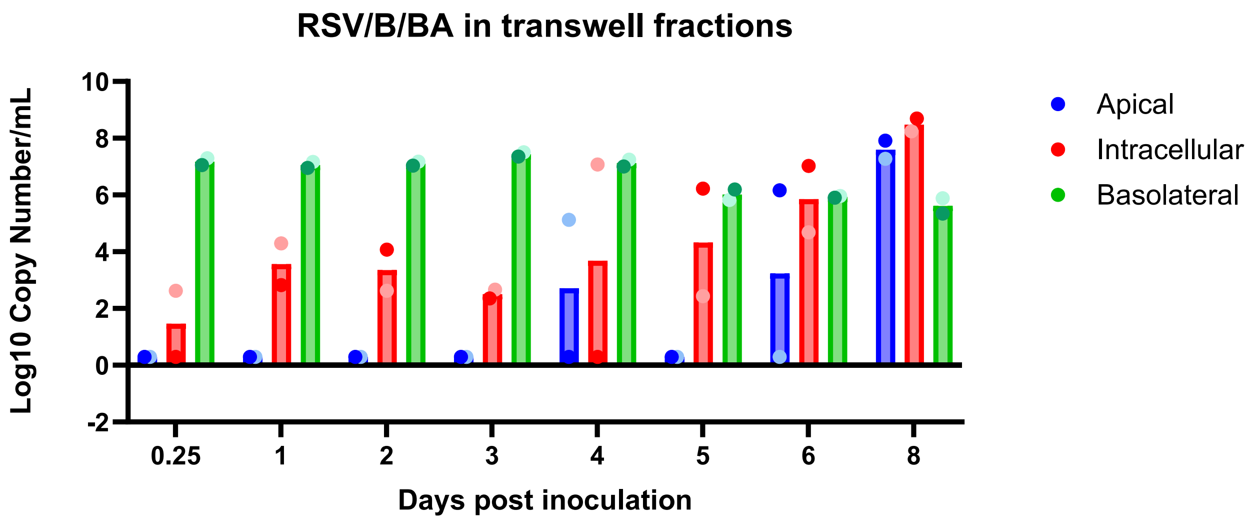

Supplemental Figure 4: Viral RNA in apical, intracellular, and basolateral compartments. qPCR of apical, intracellular, and basolateral viral RNA with basolateral inoculation of RSV/B/BA in a single adult differentiated HNO-ALI line. Data represent mean of duplicate transwells/timepoint. Dark and light-colored data points are matched samples from the same transwell.

Supplemental Figure 5

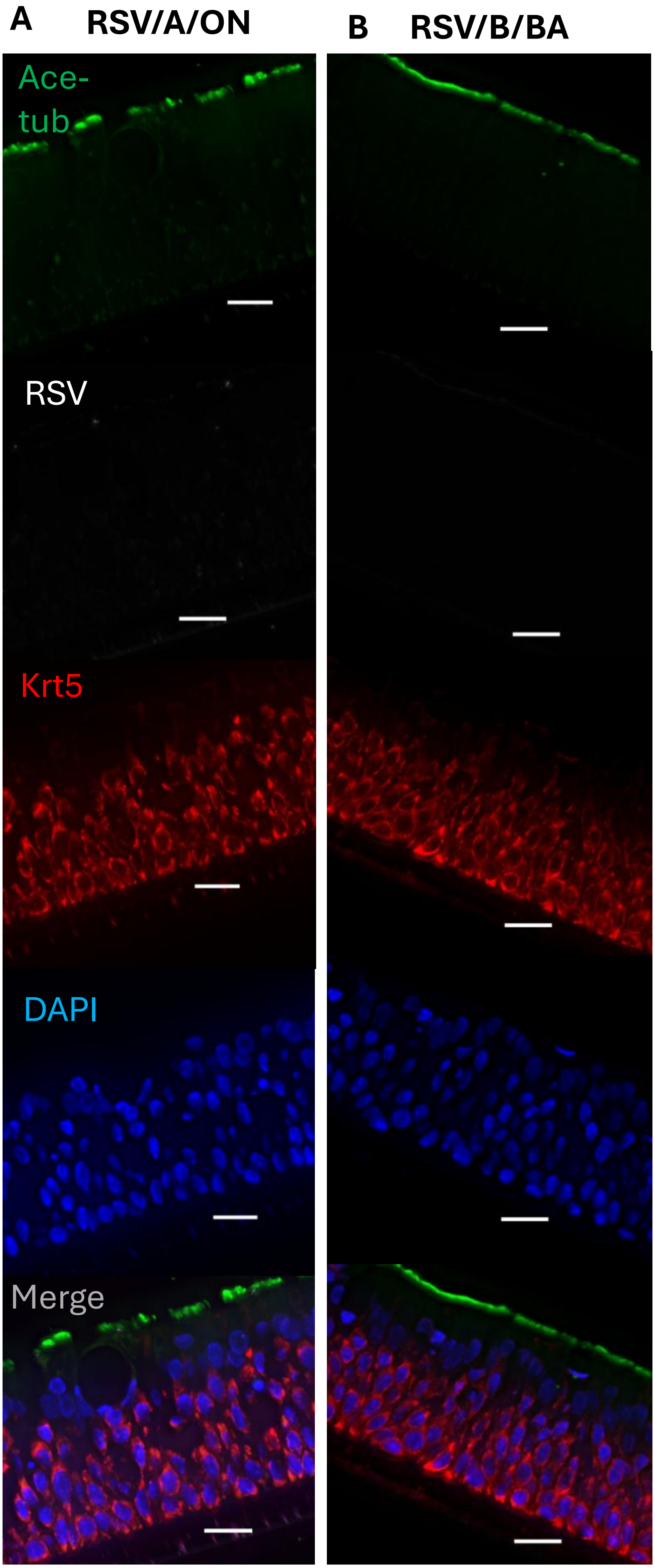

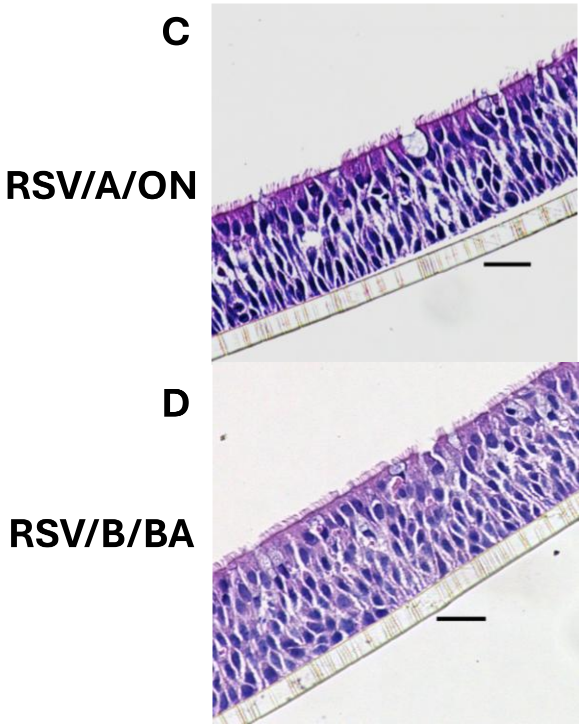

Supplemental Figure 5: RSV/A/ON and RSV/B/BA basolateral inoculation of HNO-ALIs with no evidence of infection or epithelial damage. Representative IF images of a single representative infant HNO-ALI line basolaterally inoculated with (A) RSV/A/ON or (B) RSV/B/BA at 8 dpi. Ciliated cells – Acetylated alpha-tubulin 'Ace-tub' (green), RSV (white), Basal cells - Krt5(red), and nuclei – DAPI (blue). Representative H&E images of a single infant HNO-ALI line basolaterally inoculated with (C) RSV/A/ON or (D) RSV/B/BA at 8 dpi. Scale bar is 20 μm.

Supplemental Figure 6

Basolateral Infection

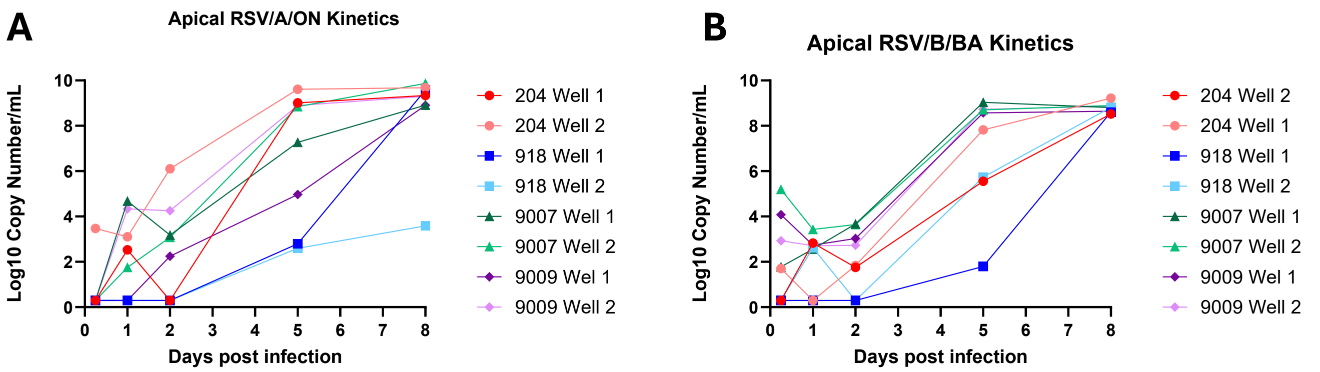

Apical Infection

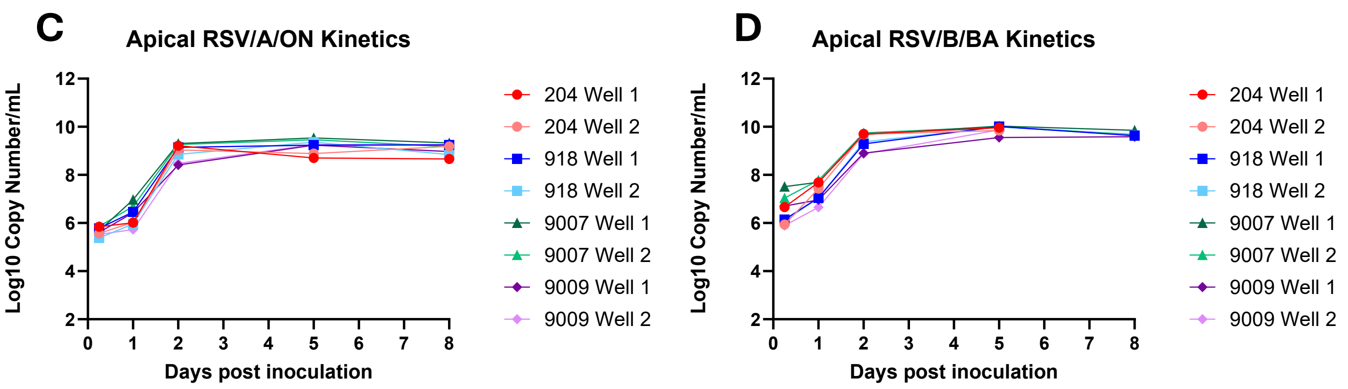

Supplemental Figure 6: Apical detection of RSV/A/ON and RSV/B/BA with simultaneous TEER monitoring. qPCR of apical viral RNA from (A-B) basolateral and (C-D) apical inoculation of adult (204 and 918) and infant (9007 and 9009) HNO-ALIs with RSV/A/ON and RSV/B/BA.

Supplemental Figure 7

A

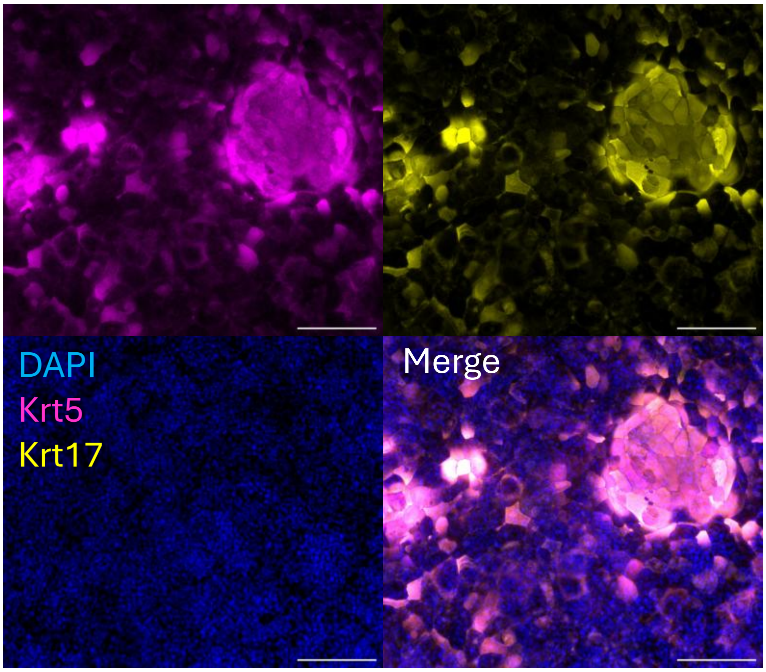

B

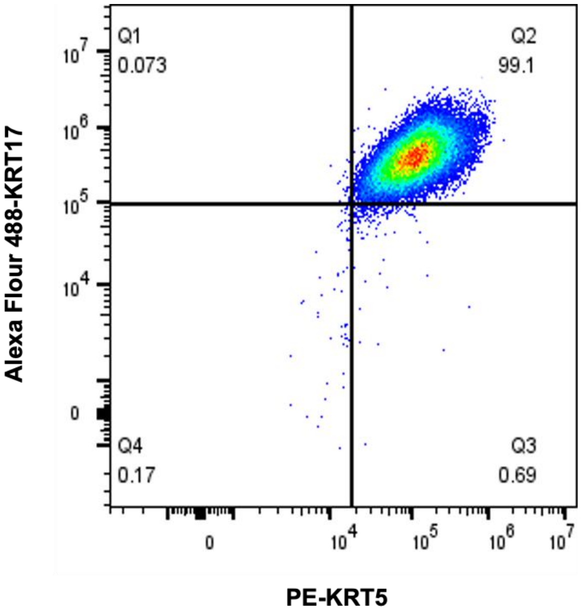

Supplemental Figure 7: Pan-basal cell markers expressed in undifferentiated HNO-ALIs. (A) Representative image of an undifferentiated adult HNO-ALI stained for Krt5 (pink), Krt17 (yellow), and DAPI (blue). (B) Co-expression of Krt5 and Krt17 in a single undifferentiated adult HNO-ALI. Flow cytometric analysis of undifferentiated adult HNO-ALI cells stained for Krt5 (x-axis) and Krt17 (y-axis).

Supplemental Figure 8

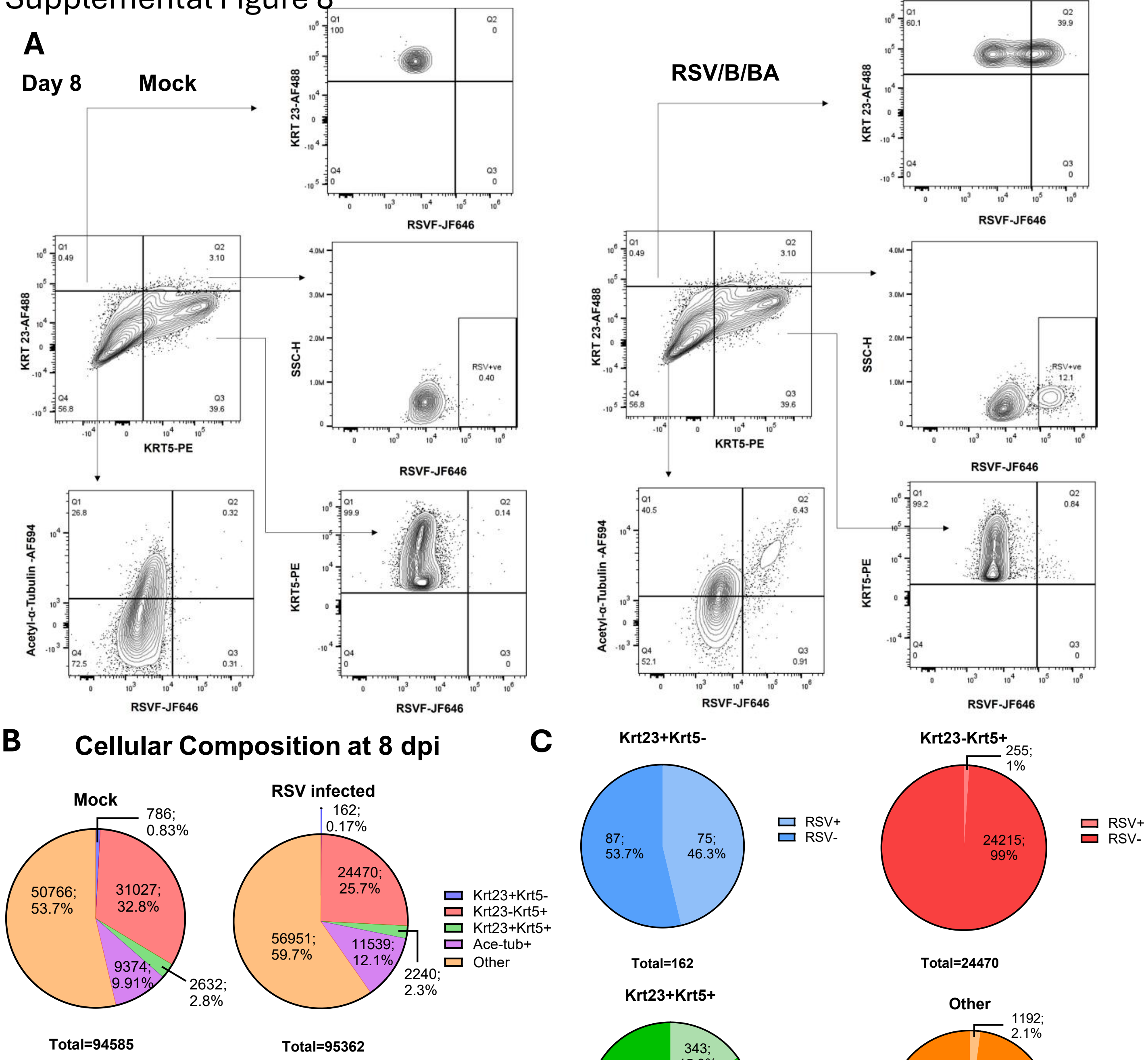

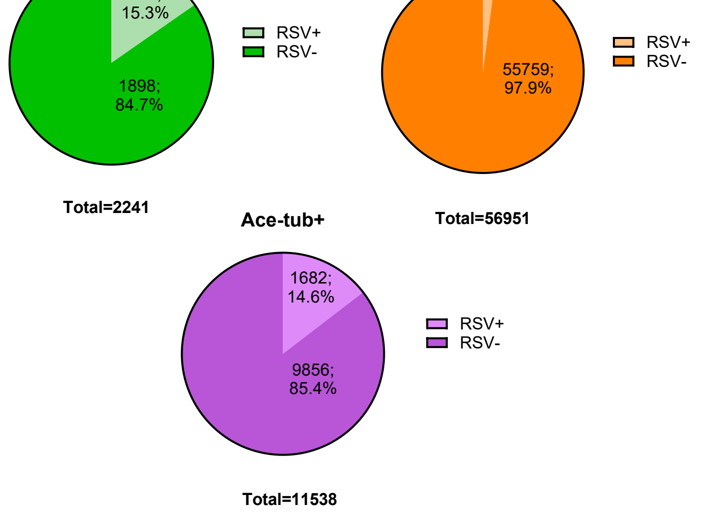

Supplemental Figure 8: RSV selectively infects Krt23+ basal cells with basolateral inoculation of HNO-ALIs at 8 dpi. (A) Representative spectral flow cytometry plots showing gating strategy to identify Krt5+, Krt23+, and Ace-tub+ cell populations in basolateral mock and RSV/B/BA inoculated HNO-ALIs at 8 dpi. Further gating of RSV F protein was used to determine RSV infection in each cell population**.** (B) Pie charts summarizing the average proportion of each cell population in basolateral mock and RSV/B/BA inoculated HNO-ALIs at 8 dpi. (C) Pie charts summarizing RSV/B/BA infection in each cell population at 8 dpi.
